# Low Cholesterol is Associated With Mortality From Stroke, Heart Disease, and Cancer: The Jichi Medical School Cohort Study

**DOI:** 10.2188/jea.JE20100065

**Published:** 2011-01-05

**Authors:** Naoki Nago, Shizukiyo Ishikawa, Tadao Goto, Kazunori Kayaba

**Affiliations:** 1 Tokyo-kita Social Health Insurance Hospital, Clinical Education Center, Tokyo, Japan; 2 Jichi Medical University, Tochigi, Japan; 3 Wara National Health Insurance Clinic, Gifu, Japan; 4 Saitama Prefectural University, Saitama, Japan

**Keywords:** low cholesterol, mortality, liver disease, stroke, heart disease, cohort study

## Abstract

**Background:**

We investigated the relationship between low cholesterol and mortality and examined whether that relationship differs with respect to cause of death.

**Methods:**

A community-based prospective cohort study was conducted in 12 rural areas in Japan. The study subjects were 12 334 healthy adults aged 40 to 69 years who underwent a mass screening examination. Serum total cholesterol was measured by an enzymatic method. The outcome was total mortality, by sex and cause of death. Information regarding cause of death was obtained from death certificates, and the average follow-up period was 11.9 years.

**Results:**

As compared with a moderate cholesterol level (4.14–5.17 mmol/L), the age-adjusted hazard ratio (HR) of low cholesterol (<4.14 mmol/L) for mortality was 1.49 (95% confidence interval [CI]: 1.23–1.79) in men and 1.50 (1.10–2.04) in women. High cholesterol (≥6.21 mmol/L) was not a risk factor. This association was unchanged in analyses that excluded deaths due to liver disease, which yielded age-adjusted HRs of 1.38 (95% CI, 1.13–1.67) in men and 1.49 (1.09–2.04) in women. The multivariate-adjusted HRs and 95% CIs of the lowest cholesterol group for hemorrhagic stroke, heart failure (excluding myocardial infarction), and cancer mortality significantly higher than those of the moderate cholesterol group, for each cause of death.

**Conclusions:**

Low cholesterol was related to high mortality even after excluding deaths due to liver disease from the analysis. High cholesterol was not a risk factor for mortality.

## INTRODUCTION

Both medical professionals and patients are well aware of the dangers of high cholesterol, but most know little about the risks of low cholesterol, despite the many studies that have examined the issue.^1^^–^^3^ The first report to show a relationship between low cholesterol and cerebral hemorrhage was a Japanese cohort study,^4^ and many subsequent observational studies have shown that low cholesterol is associated with cerebral hemorrhage,^5^ cancer, suicide, injury, and non-coronary mortality.^6^^–^^8^ However, there is no explicit evidence that these relationships are causal.

A meta-analysis of interventional trials showed that cholesterol-lowering therapy was associated with high mortality in a population with low cardiovascular risk.^9^ Although this meta-analysis focused on interventions other than statins, studies of statins have also shown that statin administration is associated with increases in cancer incidence among elderly adults,^10^ breast cancer incidence during the secondary prevention phase,^11^ and total cancer incidence.^12^

Japanese researchers reported that the relationship between low cholesterol and mortality disappeared when deaths due to liver disease were excluded.^13^

To clarify this issue, we investigated the relationship between cholesterol and mortality with respect to cause of death (deaths due to stroke, heart disease, and cancer). In addition, the relationship between cholesterol and mortality was examined after excluding deaths due to liver disease.

## METHODS

### Participants

This study was conducted as part of the Jichi Medical School (JMS) Cohort Study^13^; 12 490 men and women aged 40 to 69 years participated. The JMS Cohort Study is a prospective cohort study that began in 1992 with the aim of investigating risk factors for stroke and cardiovascular diseases. We collected baseline data from April 1992 through July 1995 in 12 rural areas of Japan and completed a follow-up in December 2005, in which 65% of the subjects from mass screening examinations participated. Of these, 96% of participants completed follow-up; incomplete data were obtained for 409 participants who were not followed until the last day of our study because they had left the study area. There were no follow-up data for 97 participants. However, data from the abovementioned 409 subjects were included in the analyses, and the day they left the area was defined as the endpoint. Informed consent for follow-up was not obtained from 95 participants and from 2 additional participants who had already left the area at the beginning of follow-up. These 97 individuals were excluded from the analyses. Furthermore, we were unable to obtain total cholesterol data for 156 participants, including 4 who did not provide informed consent. In total, we analyzed data from 12 241 participants, ie, 98% of the total number of participants. The average follow-up period was 11.9 years. We observed participants for a total of 145 312 person-years. Details regarding the JMS Cohort Study are available elsewhere.^14^

### Exposure

Total cholesterol was measured by an enzymatic method (Wako, Osaka, Japan; interassay coefficient of variation: 1.5%). All samples were measured at the same laboratory (SRL, Tokyo, Japan).

### Confounding factors

We obtained information on confounding factors (smoking and drinking habits, blood pressure, height, weight, and high-density lipoprotein [HDL] cholesterol) from the baseline data of the JMS Cohort Study.

### Outcome

Information regarding cause of death was collected using data from death certificates and national vital statistics with the permission of the Agency of General Affairs.

We classified cause of death according to the International Classification of Diseases, 10th Revision (hemorrhagic stroke: I60, I61, I69.0, I69.1; ischemic stroke: I63, I69.3; myocardial infarction: I21, I22; heart failure: I50).

Written informed consent was obtained from all participants. The Institutional Review Board of JMS was responsible for ethical review of this research and approved the study.

### Statistical analysis

Because the relationship between cholesterol and mortality is not linear, subjects were divided into 4 groups according to total cholesterol level (<4.14 mmol/L, 4.14 mmol/L to <5.17 mmol/L, 5.17 mmol/L to <6.21 mmol/L, and ≥6.21 mmol/L). The second lowest group (4.14 mmol/L to <5.17 mmol/L) was used as the reference group. However, in the analyses of ischemic stroke in men and myocardial infarction in women, the highest group was defined as ≥5.70 mmol/L because there was no ischemic stroke or myocardial infarction in subjects with a total cholesterol level ≥6.21 mmol/L. The cutoff value used for the lowest group was selected on the basis of past research,^1^ and the cutoff value for the highest group was based on criteria from the mass screening examination.

A Cox proportional hazards model was used to calculate hazard ratios (HRs) and 95% confidence intervals (CIs) for mortality. Both age-adjusted HRs and multivariate-adjusted HRs were calculated. The multivariate-adjusted model adjusted for age, smoking status (current/former/never), drinking status (current/former/never), systolic blood pressure, HDL cholesterol, and body mass index (BMI:<18, 18 to <22, 22 to <26, ≥26). Age, blood pressure, and HDL cholesterol were analyzed as continuous variables. All analyses were performed separately for each sex using STATA/SE for Windows (STATACORP, release 10, TX, USA). A *P* value of less than 0.05 was considered to indicate statistical significance.

## RESULTS

All subjects were included in the age-adjusted analysis. Because of missing values, multivariate-adjusted analyses included data from only 11 869 subjects. Tables 1 and 2 show the baseline characteristics of subjects grouped by sex and cholesterol category. The background characteristics of subjects included and excluded from the multivariate-adjusted analysis did not differ.

**Table 1.** Baseline characteristics of participants

Variable	Men			Women		
	
	*n*	Mean	SD	*n*	Mean	SD
Age	4839	55.2	12	7495	55.3	11.3
Height (cm)	4649	162.5	6.9	7250	150.3	6.2
Body weight (kg)	4651	60.9	9.4	7252	52.4	8.0
Systolic BP (mm Hg)	4664	131.5	20.5	7297	128.3	21.0
Diastolic BP (mm Hg)	4664	79.2	12.3	7297	76.3	12.1
Total cholesterol (mmol/L)	4839	4.8	0.9	7495	5.1	0.9
HDL cholesterol (mmol/L)	4839	1.3	0.4	7495	1.4	0.3
Body mass index (kg/m^2^)	4649	23.0	2.9	7250	23.2	3.2

Smoking	*n*	%		*n*	%	
		
Current	2280	50.5		384	5.5	
Former	1274	28.2		195	2.8	
Never	957	21.2		6362	91.7	
Drinking						
Current	3293	75.0		1692	25.0	
Former	161	3.7		103	1.5	
Never	935	21.3		4983	73.5	

Abbreviations: BP, blood pressure; HDL, high-density lipoprotein.

**Table 2.** Baseline characteristics of participants by cholesterol level

**Men**									

	Variable	Total cholesterol level (mmol/L)								*P*^a^
	
		<4.14		4.14–5.16		5.17–6.20		≥6.21		
				
		*n*	Mean (SD)	*n*	Mean (SD)	*n*	Mean (SD)	*n*	Mean (SD)	

Age		1136	55.3 (13.2)	2142	55.6 (11.8)	1274	54.9 (11.5)	287	53.0 (10.7)	0.038
Height (cm)		1079	162.4 (7.2)	2068	162.3 (6.8)	1225	162.8 (6.9)	277	163.4 (6.4)	<.0001
Body weight (kg)		1081	58.6 (9.0)	2068	60.5 (9.1)	1225	62.8 (9.6)	277	64.3 (9.8)	<.0001
Systolic BP (mm Hg)		1095	129.0 (20.9)	2069	131.3 (20.2)	1224	133.4 (20.5)	276	134.5 (20.4)	<.0001
Diastolic BP (mm Hg)		1095	77.2 (12.1)	2069	79.1 (12.1)	1224	80.8 (12.4)	276	81.3 (12.0)	<.0001
HDL cholesterol (mmol/L)		1136	1.2 (0.3)	2142	1.3 (0.3)	1274	1.3 (0.4)	287	1.3 (0.4)	<.0001
Body mass index (kg/m^2^)		1079	22.2 (2.7)	2068	22.9 (2.8)	1225	23.6 (3.0)	277	24 (2.9)	<.0001

		*n*	%	*n*	%	*n*	%	*n*	%	*P*^b^
						
Smoking	Current	627	59.1	986	49.3	532	45.2	135	49.5	<.0001
	Former	227	21.4	579	29.0	389	33.1	79	28.9	
	Never	207	19.5	435	21.8	256	21.8	59	21.6	

Drinking	Current	760	74.4	1461	75.1	871	75.6	201	74.4	0.628
	Former	47	4.6	66	3.4	41	3.6	7	2.6	
	Never	215	21.0	418	21.5	240	20.8	62	23.0	


**Women**									

	Variable	Total cholesterol level (mmol/L)								*P*^a^
	
		<4.14		4.14–5.16		5.17–6.20		≥6.21		
				
		*n*	Mean (SD)	*n*	Mean (SD)	*n*	Mean (SD)	*n*	Mean (SD)	

Age		1077	48.2 (12.7)	3050	54.4 (11.7)	2522	58.3 (9.4)	846	59.1 (7.9)	<.0001
Height (cm)		1012	152.0 (6.7)	2944	150.7 (6.2)	2463	149.4 (5.9)	831	149.3 (5.5)	<.0001
Body weight (kg)		1012	51.4 (7.7)	2944	52.2 (8.0)	2463	52.7 (8.1)	831	53.2 (7.9)	<.0001
Systolic BP^a^ (mm Hg)		1030	118.8 (18.8)	2961	126.6 (20.7)	2471	131.8 (20.9)	835	135.6 (20.4)	<.0001
Diastolic BP^a^ (mm Hg)		1030	71.2 (11.5)	2961	75.4 (11.8)	2471	78.2 (11.9)	835	80.4 (11.9)	<.0001
HDL cholesterol (mmol/L)		1077	1.2 (0.3)	3050	1.4 (0.3)	2522	1.4 (0.3)	846	1.4 (0.4)	<.0001
Body mass index (kg/m^2^)		1012	22.3 (3.1)	2944	23 (3.1)	2463	23.6 (3.2)	831	23.8 (3.1)	<.0001

		*n*	%	*n*	%	*n*	%	*n*	%	*P*^b^
						
Smoking	Current	79	8.1	171	6.0	100	4.3	39	4.3	<.0001
	Former	48	4.9	76	2.7	53	2.3	19	2.3	
	Never	853	87.0	2591	91.3	2184	93.5	790	93.5	

Drinking	Current	289	30.5	730	26.5	516	22.5	177	21.1	<.0001
	Former	14	1.5	34	1.2	45	2.0	10	1.2	
	Never	644	68.0	1992	72.3	1735	75.6	652	77.7	

Abbreviations: BP, blood pressure; HDL, high-density lipoprotein.

^a^One-way analysis of variance.

^b^Chi-square test.

In total, 635 men and 423 women died during the study period; 34 male deaths and 15 female deaths were due to liver diseases. Table 3a shows the number of deaths according to cause of death, including deaths due to liver disease, and incidence in each cholesterol group by sex. Liver cancer and hepatic failure were the main causes of death due to liver disease.

**Table 3a.** Causes of death

Cause of death	Men		Women	
	
	*n*	%	*n*	%
Stroke	67	10.6	60	14.4
SAH	7	1.1	17	4.0
infarction	36	5.7	28	6.6
hemorrhage	20	3.1	13	3.0
other	4	0.6	2	0.5
Heart disease	73	11.5	60	14.2
infarction	31	4.9	27	6.0
heart failure	20	3.1	13	3.0
other	22	3.5	20	4.7
Cancer^a^	240	37.9	154	36.0
lung	69	10.9	21	5.0
stomach	26	4.1	22	5.0
colon	17	2.7	18	4.2
other	128	20.1	93	22.0
Other	255	40.1	149	34.7
pneumonia	67	10.6	25	5.9
suicide	24	3.8	17	4.0
accident	30	4.7	7	1.7
other	133	20.9	100	23.6

Total	635	100	423	100

Liver disease				
cancer	16	47.1	3	20.0
liver failure	14	41.2	11	73.3
other	4	11.7	1	6.7

Total	34	100	15	100

Abbreviation: SAH, subarachnoid hemorrhage.

^a^Including liver cancer.

**Table 3b.** Mortality by total cholesterol level

	Total cholesterol level (mmol/L)							

	<4.14		4.14–5.16		5.17–6.20		≥6.21	
			
	*n*	incidence^a^	*n*	incidence	*n*	incidence	*n*	incidence
**Men**								
Total mortality	195	15.08	258	10.27	152	10.10	30	8.77
Stroke mortality	19	1.47	29	1.15	16	1.06	3	0.88
Hemorrhagic stroke	10	0.77	10	0.40	4	0.27	2	0.58
Ischemic stroke	7	0.54	17	0.68	12	0.80	0	0.00
Heart disease mortality	21	1.62	22	0.88	15	1.00	4	1.17
Myocardial infarction	9	0.70	13	0.52	6	0.40	2	0.58
Heart failure^b^	5	0.39	6	0.24	6	0.40	2	0.58
Cancer mortality	80	6.19	91	3.62	59	3.92	10	2.92

**Women**								
Total mortality	53	4.25	169	4.68	151	5.01	50	4.95
Stroke mortality	6	0.48	21	0.58	24	0.80	9	0.89
Hemorrhagic stroke	5	0.40	6	0.17	13	0.43	5	0.50
Ischemic stroke	1	0.08	12	0.33	11	0.37	4	0.40
Heart disease mortality	7	0.56	26	0.72	17	0.56	5	0.50
Myocardial infarction	3	0.24	15	0.42	9	0.30	0	0.00
Heart failure^b^	4	0.32	3	0.08	2	0.07	3	0.30
Cancer mortality	19	1.52	53	1.47	56	1.86	26	2.58

^a^per 1000 person-years.

^b^excluding myocardial infarction.

Table 4a shows HRs and 95% CIs for mortality by cholesterol category. Crude, age-adjusted, and multivariate-adjusted HRs are grouped by sex. Smoking status, drinking status, and BMI were entered into the models as categorical dummy variables. The multivariate-adjusted HR of the lowest cholesterol group (<4.14 mmol/L) was 1.38 (95% CI, 1.13–1.66) for men and 1.42 (1.02–2.00) for women. The HRs of men and women in the highest cholesterol group (≥6.21 mmol/L) were not significant; the HR was less than 1 for women (HR, 0.93; 95% CI, 0.67–1.30).

**Table 4a.** Total cholesterol level and mortality

	Total cholesterol level (mmol/L)			
	
	<4.14	4.14–5.16	5.17–6.20	≥6.21
	Hazard ratio (95% CI)			
**Men**				
Total mortality				
Crude	1.48 (1.23–1.78)	1	0.98 (0.80–1.20)	0.85 (0.58–1.23)
Age-adjusted	1.49 (1.23–1.79)	1	1.04 (0.85–1.27)	1.12 (0.77–1.64)
Multivariate- ​ adjusted^a^	1.38 (1.13–1.66)	1	1.09 (0.88–1.34)	1.21 (0.82–1.78)

Excluding deaths due to liver disease		
Crude	1.37 (1.13–1.66)	1	0.99 (0.81–1.22)	0.87 (0.60–1.28)
Age-adjusted	1.38 (1.13–1.67)	1	1.06 (0.86–1.29)	1.16 (0.80–1.71)
Multivariate- ​ adjusted^a^	1.27 (1.03–1.56)	1	1.10 (0.89–1.36)	1.25 (0.85–1.85)

**Women**				
Total mortality				
Crude	0.92 (0.67–1.25)	1	1.07 (0.86–1.33)	1.06 (0.77–1.45)
Age-adjusted	1.50 (1.10–2.04)	1	0.92 (0.74–1.14)	0.97 (0.67–1.26)
Multivariate- ​ adjusted^a^	1.42 (1.02–2.00)	1	0.93 (0.73–1.17)	0.93 (0.67–1.30)

Excluding deaths due to liver disease		
Crude	0.91 (0.66–1.24)	1	1.06 (0.85–1.32)	1.04 (0.76–1.44)
Age-adjusted	1.49 (1.09–2.04)	1	0.91 (0.72–1.14)	0.91 (0.66–1.26)
Multivariate- ​ adjusted^a^	1.40 (0.99–1.98)	1	0.92 (0.72–1.17)	0.93 (0.66–1.31)

^a^Cox proportional hazards model adjusted for age, systolic blood pressure, high-density lipoprotein cholesterol, smoking, drinking, and body mass index.

Among women, there was an inverse association between total cholesterol and mortality age-adjusted analysis. The multifactor-adjusted HR in the lowest cholesterol group was 0.50 (95% CI, 0.16–1.55) in premenopausal women and 1.42 (0.98–2.06) in postmenopausal women.

Table 4a shows the same results, after excluding deaths due to liver disease. In these analyses, the age-adjusted HRs of the lowest cholesterol group were statistically significant: 1.38 (1.13–1.67) for men and 1.49 (1.09–2.04) for women. There was no difference between these results and those for all causes of mortality.

The results of analyses that excluded deaths within the first 5 years of follow-up were similar to those that included all deaths (Table 4b). In addition, the results of analysis that excluded 320 participants with a history of stroke (113), myocardial infarction (65), or cancer (142) were similar (Table 4c).

**Table 4b.** Total cholesterol and mortality (excluding deaths within the first 5 years of follow-up)

	Total cholesterol level (mmol/L)			
	
	<4.14	4.14–5.16	5.17–6.20	≥6.21
	Hazard ratio (95% CI)			
**Men**				
Total mortality				
Crude	1.43 (1.16–1.79)	1	1.01 (0.80–1.27)	0.91 (0.60–1.40)
Age-adjusted	1.47 (1.18–1.83)	1	1.07 (0.85–1.35)	1.24 (0.81–1.89)
Multivariate- ​ adjusted^a^	1.39 (1.10–1.74)	1	1.07 (0.84–1.37)	1.33 (0.87–2.05)

Excluding deaths due to liver disease		
Crude	1.33 (1.06–1.68)	1	1.03 (0.82–1.30)	0.94 (0.62–1.44)
Age-adjusted	1.36 (1.08–1.71)	1	1.09 (0.87–1.38)	1.28 (0.84–1.97)
Multivariate- ​ adjusted^a^	1.27 (1.00–1.62)	1	1.11 (0.87–1.41)	1.39 (0.90–2.14)

**Women**				
Total mortality				
Crude	0.82 (0.57–1.20)	1	0.99 (0.77–1.29)	1.14 (0.80–1.63)
Age-adjusted	1.37 (0.94–2.00)	1	0.86 (0.66–1.11)	0.99 (0.70–1.43)
Multivariate- ​ adjusted^a^	1.24 (0.82–1.88)	1	0.92 (0.70–1.22)	1.11 (0.76–1.61)

Excluding deaths due to liver disease		
Crude	0.80 (0.55–1.18)	1	0.99 (0.76–1.30)	1.15 (0.80–1.65)
Age-adjusted	1.34 (0.91–1.98)	1	0.86 (0.66–1.12)	0.91 (0.66–1.26)
Multivariate- ​ adjusted^a^	1.20 (0.79–1.84)	1	0.92 (0.70–1.23)	0.93 (0.66–1.31)

^a^Cox proportional hazards model adjusted for age, systolic blood pressure, high-density lipoprotein cholesterol, smoking, drinking, and body mass index.

**Table 4c.** Total cholesterol and mortality (excluding participants with a history of cancer, stroke, or myocardial infarction)

	Total cholesterol levels (mmol/L)			
	
	<4.14	4.14–5.16	5.17–6.20	≥6.21
	Hazard ratio (95% CI)			
**Men**				
Total mortality				
Crude	1.49 (1.21–1.83)	1	0.96 (0.76–1.19)	0.87 (0.60–1.27)
Age-adjusted	1.48 (1.21–1.82)	1	1.02 (0.81–1.27)	1.06 (0.73–1.55)
Multivariate- ​ adjusted^a^	1.38 (1.12–1.71)	1	1.08 (0.86–1.36)	1.25 (0.83–1.87)

Excluding deaths due to liver disease		
Crude	1.39 (1.12–1.72)	1	0.98 (0.78–1.23)	0.91 (0.62–1.33)
Age-adjusted	1.38 (1.13–1.71)	1	1.04 (0.83–1.31)	1.11 (0.76–1.62)
Multivariate- ​ adjusted^a^	1.29 (1.03–1.60)	1	1.10 (0.88–1.39)	1.30 (0.87–1.94)

**Women**				
Total mortality				
Crude	0.77 (0.53–1.12)	1	1.16 (0.92–1.47)	1.09 (0.78–1.53)
Age-adjusted	1.29 (0.89–1.88)	1	0.96 (0.76–1.22)	0.90 (0.64–1.26)
Multivariate- ​ adjusted^a^	1.28 (0.88–1.89)	1	0.99 (0.78–1.27)	0.90 (0.63–1.30)

Excluding deaths due to liver disease		
Crude	0.78 (0.53–1.14)	1	1.15 (0.90–1.47)	1.08 (0.77–1.53)
Age-adjusted	1.30 (0.89–1.91)	1	0.95 (0.75–1.22)	0.89 (0.63–1.26)
Multivariate- ​ adjusted^a^	1.29 (0.88–1.91)	1	0.99 (0.77–1.27)	0.90 (0.62–1.30)

^a^Cox proportional hazards model adjusted for age, systolic blood pressure, high-density lipoprotein cholesterol, smoking, drinking, and body mass index.

Table 5 shows HRs and 95% CIs for stroke, heart disease, and cancer mortality according to cholesterol category. The multivariate-adjusted HR of the lowest cholesterol group was higher than 1 for each cause of death and was statistically significant for cancer mortality in men.

**Table 5.** Total cholesterol by cause of death

	Total cholesterol level (mmol/L)			
	
	<4.14	4.14–5.16	5.17–6.21	≥6.21
	Hazard ratio (95% CI)			
**Men**				
Stroke mortality				
Crude	1.23 (0.72–2.27)	1	0.92 (0.50–1.69)	0.75 (0.23–2.49)
Age-adjusted	1.28 (0.72–2.28)	1	0.98 (0.53–1.80)	1.02 (0.31–3.34)
Multivariate-adjusted^a^	1.21 (0.66–2.21)	1	0.99 (0.53–1.82)	0.98 (0.29–3.23)
Hemorrhagic stroke				
Crude	1.93 (0.81–4.62)	1	0.65 (0.21–2.01)	1.79 (0.50–6.45)
Age-adjusted	1.92 (0.80–4.61)	1	0.69 (0.22–2.19)	2.10 (0.58–7.61)
Multivariate-adjusted^a^	1.96 (0.80–4.79)	1	0.68 (0.21–2.16)	1.76 (0.38–8.09)
Ischemic stroke^b^				
Crude	0.84 (0.35–2.04)	1	1.56 (0.71–3.40)	0.48 (0.14–1.64)
Age-adjusted	0.85 (0.35–2.06)	1	1.59 (0.73–3.47)	0.58 (0.17–1.95)
Multivariate-adjusted^a^	0.79 (0.30–2.04)	1	1.55 (0.70–3.43)	0.65 (0.19–2.23)
Heart disease mortality				
Crude	1.74 (1.00–3.01)	1	1.06 (0.58–1.93)	1.36 (0.54–3.53)
Age-adjusted	1.75 (1.01–3.04)	1	1.14 (0.62–2.10)	1.93 (0.74–5.03)
Multivariate-adjusted^a^	1.36 (0.76–2.46)	1	1.04 (0.54–2.01)	2.14 (0.81–5.65)
Myocardial infarction				
Crude	1.34 (0.57–3.12)	1	0.76 (0.29–1.99)	1.38 (0.40–4.82)
Age-adjusted	1.34 (0.58–3.15)	1	0.84 (0.32–2.19)	1.84 (0.53–6.483)
Multivariate-adjusted^a^	0.99 (0.40–2.46)	1	0.86 (0.30–2.47)	2.37 (0.52–10.83)
Heart failure^c^				
Crude	1.63 (0.50–5.32)	1	1.64 (0.54–5.01)	1.94 (0.40–9.40)
Age-adjusted	1.72 (0.52–5.66)	1	1.90 (0.61–5.89)	2.77 (0.56–13.67)
Multivariate-adjusted^a^	1.32 (0.36–4.79)	1	1.59 (0.48–5.27)	3.86 (0.76–19.58)
Cancer mortality				
Crude	1.72 (1.27–2.32)	1	1.08 (0.78–1.49)	0.80 (0.42–1.53)
Age-adjusted	1.73 (1.28–2.34)	1	1.13 (0.82–1.57)	1.01 (0.53–1.95)
Multivariate-adjusted^a^	1.66 (1.22–2.27)	1	1.18 (0.85–1.66)	1.07 (0.55–2.07)

**Women**				
Stroke mortality				
Crude	0.83 (0.33–2.05)	1	1.37 (0.76–2.46)	1.53 (0.70–3.35)
Age-adjusted	1.41 (0.57–3.50)	1	1.19 (0.66–2.15)	1.40 (0.64–3.06)
Multivariate-adjusted^a^	1.84 (0.71–4.76)	1	1.29 (0.68–2.44)	1.52 (0.66–3.48)
Hemorrhagic stroke				
Crude	2.18 (0.70–6.81)	1	2.22 (0.92–5.37)	3.28 (1.21–8.87)
Age-adjusted	3.41 (1.07–10.85)	1	1.97 (0.80–4.85)	2.92 (1.05–8.07)
Multivariate-adjusted^a^	3.86 (1.18–12.68)	1	1.94 (0.77–4.89)	2.15 (0.68–6.77)
Ischemic stroke				
Crude	0.23 (0.03–1.81)	1	1.06 (0.48–2.39)	1.08 (0.35–3.28)
Age-adjusted	0.44 (0.06–3.39)	1	0.99 (0.44–2.23)	1.06 (0.34–3.28)
Multivariate-adjusted^a^	0.57 (0.07–4.54)	1	0.90 (0.37–2.19)	1.17 (0.36–3.85)
Heart disease mortality				
Crude	0.75 (0.33–1.73)	1	0.93 (0.53–1.65)	0.66 (0.26–1.73)
Age-adjusted	1.43 (0.62–3.40)	1	0.87 (0.50–1.55)	0.69 (0.26–1.80)
Multivariate-adjusted^a^	1.34 (0.54–3.35)	1	0.78 (0.42–1.42)	0.39 (0.11–1.30)
Myocardial infarction^b^				
Crude	0.58 (0.17–2.01)	1	0.51 (0.17–1.53)	0.53 (0.20–1.46)
Age-adjusted	1.09 (0.31–3.78)	1	0.50 (0.16–1.49)	0.49 (0.18–1.36)
Multivariate-adjusted^a^	1.07 (0.30–3.79)	1	0.38 (0.11–1.34)	0.52 (0.18–1.46)
Heart failure^c^				
Crude	3.75 (0.87–16.19)	1	0.71 (0.13–3.99)	4.22 (1.00–17.82)
Age-adjusted	6.57 (1.49–28.97)	1	0.66 (0.11–3.77)	4.00 (0.92–17.45)
Multivariate-adjusted^a^	5.79 (1.07–31.27)	1	0.72 (0.12–4.28)	2.33 (0.37–14.66)
Cancer mortality				
Crude	1.04 (0.62–1.76)	1	1.26 (0.87–1.84)	1.75 (1.09–2.80)
Age-adjusted	1.50 (0.89–2.55)	1	1.09 (0.75–1.58)	1.48 (0.92–2.36)
Multivariate-adjusted^a^	1.44 (0.83–2.49)	1	1.07 (0.72–1.59)	1.58 (0.97–2.56)

^a^Cox proportional hazards model adjusted for age, systolic blood pressure, high-density lipoprotein cholesterol, smoking, drinking, and body mass index.

^b^Cholesterol levels: <4.13, 4.14–5.16, 5.17–5.69, ≥5.70.

^c^Excluding myocardial infarction.

We separately analyzed participants with and without ischemia for stroke and heart disease. Among subjects with the highest level of total cholesterol, the multivariate-adjusted HRs for ischemic stroke and myocardial infarction were not significant. The corresponding HRs for ischemic stroke in men and myocardial infarction in women were less than 1; however, the HR of the lowest group was 3.86 (95% CI, 1.18–12.68) for hemorrhagic stroke in women and 5.79 (1.07–31.27) for heart failure excluding myocardial infarction in women.

## DISCUSSION

We noted a clear relationship between low cholesterol and increased mortality. Okamura et al^13^ reported that occult liver diseases are associated with mortality; however, in the present study, the relationship between low cholesterol and increased mortality was unchanged in analyses that excluded deaths due to liver disease. Our results suggest that hemorrhagic stroke and heart failure excluding myocardial infarction, contribute to the relationship between low cholesterol and high mortality.

Studies have shown a relationship between low cholesterol and non-cardiovascular mortality; however, in addition to cancer mortality, stroke mortality and heart disease mortality were also related to low cholesterol in our analyses. The relationship between low cholesterol and hemorrhagic stroke was similar to previously reported results.^3^^,^^5^ Although the relationship between high cholesterol and ischemic stroke is not constant, it may be that the risk of high cholesterol disappears due to medical interventions for ischemic stroke and that the risk of low cholesterol is thus emphasized because of a lack of such interventions for low cholesterol.

It is difficult to interpret the relationship between low cholesterol and heart disease mortality. Although a relationship between cholesterol and heart failure was reported, high cholesterol, too, was a risk factor for non-ischemic heart failure.^16^ We found no report of an association between low cholesterol and heart failure. The effects of malnutrition should be considered, as should the possible presence of beriberi heart disease and alcoholism. The relationship between low cholesterol and heart disease mortality was stronger in women than in men, so a disease like hyperthyroidism, which is more common in women, may be the culprit. A meta-analysis found an increase in cardiovascular mortality associated with subclinical hyperthyroidism,^17^ but further investigations are necessary to confirm this hypothesis.

Because low cholesterol is associated with high cancer mortality, low cholesterol is a key finding in cancer. Previous studies reported an increase in liver cancer^13^^,^^15^; however, in the present study, increased cancer mortality in the lowest cholesterol group was unchanged after excluding cases of liver disease from the analyses. This suggests a need for screening of cancers other than liver cancer in individuals with low cholesterol levels.

Our results differ from those of previous studies,^1^^,^^3^ in that high cholesterol was not identified as a risk factor for mortality in the present study. The HR was 0.93 (0.67–1.30) for women, which indicates that the focus should be on adults with low cholesterol rather than those with high cholesterol.

Our analyses constitute a primary use of existing data. An important advantage of this study was that the follow-up rate was very high because the study was conducted in rural areas, where migration is far less than in urban areas.

Cause of death was ascertained using death certificates, so there were potential limitations in accuracy regarding cause of death. The setting was a periodical health examination to screen participants with high cholesterol. Thus, it is also necessary to consider the possibility that risk was underestimated due to medical therapy for high cholesterol.

Our results are specific to people living in rural areas of Japan and their lifestyle, and may not be applicable to urban Japanese or other ethnic groups. Because Japanese in rural areas have less coronary disease than people in Western countries, their risks from low cholesterol may be higher.

There are many factors that might contribute to the relationship between low cholesterol and high mortality. A correlation between high mortality and low cholesterol clearly exists, especially in populations with a low risk of coronary heart disease.^9^ Although the dangers of a high cholesterol level are widely known, they are less important in regions—such as rural Japan—where cardiovascular disease is less common. It may therefore be necessary to highlight the risks of low cholesterol. In Japan, only LDL cholesterol and HDL cholesterol are measured at present; however, total cholesterol remains an important measure in predicting mortality.

In conclusion, we observed that low cholesterol was associated with increased risks of cancer, hemorrhagic stroke, and heart failure excluding myocardial infarction.
